# Case Report: Transcatheter interventional procedure to innominate vein turn-down procedure for failing fontan circulation

**DOI:** 10.3389/fped.2024.1341443

**Published:** 2024-02-06

**Authors:** Damien Schaffner, Maria-Helena Perez, Rafael Duran, René Pretre, Stefano Di Bernardo

**Affiliations:** ^1^ Pediatric Cardiology Unit, Women Mother and Child Department, Lausanne University Hospital, Lausanne, Switzerland; ^2^ Pediatric Intensive Care Unit, Women Mother and Child Department, Lausanne University Hospital, Lausanne, Switzerland; ^3^ Department of Radiology and Interventional Radiology, Lausanne University Hospital, Lausanne, Switzerland; ^4^ Department of Cardiac Surgery, Lausanne University Hospital, Lausanne, Switzerland

**Keywords:** case report, hypoplastic left heart syndrome, failing fontan, plastic bronchitis, transcatheter interventional procedure, modified fenestration

## Abstract

Fontan physiology creates a chronic state of decreased cardiac output and systemic venous congestion, leading to liver cirrhosis/malignancy, protein-losing enteropathy, chylothorax, or plastic bronchitis. Creating a fenestration improves cardiac output and relieves some venous congestion. The anatomic connection of the thoracic duct to the subclavian-jugular vein junction exposes the lymphatic system to systemic venous hypertension and could induce plastic bronchitis. To address this complication, two techniques have been developed. A surgical method that decompresses the thoracic duct by diverting the innominate vein to the atrium, and a percutaneous endovascular procedure that uses a covered stent to create an extravascular connection between the innominate vein and the left atrium. We report a novel variant transcatheter intervention of the innominate vein turn-down procedure without creating an extravascular connection in a 39-month-old patient with failing Fontan circulation complicated by plastic bronchitis and a 2-year post-intervention follow-up.

## 1 Introduction

The staged surgical palliation through total cavopulmonary anastomosis greatly improved patients’ survival with functionally univentricular cardiac anatomy to over 80% survival rate at 20 years of surgical completion (1). This circulatory setting involves a lifelong state of elevated central venous pressure. Creating a fenestration improves cardiac output and relieves some venous congestion, at the cost of decreasing oxygen saturation (2).

The anatomic connection of the thoracic duct (TD) to the subclavian-jugular vein junction exposes the lymphatic system to systemic venous hypertension. This situation induces pathophysiologic modification and causes potential lethal complications such as ascites, protein-losing enteropathy (PLE), chylothorax, or plastic bronchitis (PB). The prognosis of these complications is improved with new medical therapies and interventional approaches such as lymphatic embolization. Hraska et al. (3) described a surgical method to decompress the TD by diverting the innominate vein to the atrium. Smith et al. (4) described a transcatheter procedure using a covered stent to create an extravascular connection between the innominate vein and the left atrium. Both techniques allowed connecting the TD via the innominate vein to a physiological venous pressure system and through the usual “diastolic suctioning” to increase the lymphatic drainage/return.

We report a novel variant transcatheter intervention of the innominate vein turn-down procedure without creating an extravascular connection by a 39-month-old patient with failing Fontan circulation complicated with plastic bronchitis. The procedure was successful and we present this report after a 2-year follow-up.

## 2 Case description

A 39-month-old boy with hypoplastic left heart syndrome, large ventricular septal defect, and mitral and aortic atresia underwent a staged single ventricle palliation (Giessen I procedure) at the age of 3 days followed by a comprehensive stage II procedure at the age of 3 months. Because of decreasing transcutaneous saturation (<75%) without improvement with oral sildenafil and Bosentan introduced secondarily, one large venovenous collateral (diameter 6–9 mm) connecting the innominate vein to the coronary sinus was closed with an Amplatzer Vascular Plug II 12 mm (Abbott, Illinois, USA). This was followed, the day after, with surgical completion of Fontan circulation with a 16 mm extracardiac tube (at age 28 months). The Fontan circulation was fenestrated with a modified fenestration (Gore-Tex® 6 mm, Gore Medical, Delaware, USA) connecting the innominate vein to the right atrial (RA) appendage (5), which is done routinely in our center.

Signs of failing Fontan marked the postoperative course: an elevated mean pulmonary pressure of 18–22 mmHg and, on day 5, acute plastic bronchitis with bilateral chylothorax. In the absence of clinical improvement, despite the administration of pulmonary vasodilators, and because of critical venous congestion, it was decided to surgically clip the TD at the diaphragmatic level and to increase the fenestration size to 8 mm. Plastic bronchitis resolved and the patient was discharged with a low peripheral oxygen saturation of 75%–80% on sildenafil and Bosentan therapy as well as oral anticoagulation. The patient was listed for a heart transplant, but a severe alloimmunization HLA prevented a compatible donor.

After 10 months without plastic bronchitis, the patient relapsed with infections that triggered several bronchial cast expectorations on a daily basis, an atelectatic right upper lobe, and severe transcutaneous desaturation at 60%–65% on room air. The optimization of the conservative medical treatment with low-fat diet, high-dose spironolactone, sildenafil, Bosentan, and steroid sprays did not improve the symptomatology. Adding aerosolized tissue plasminogen activator has had no effect.

**Figure 1:**
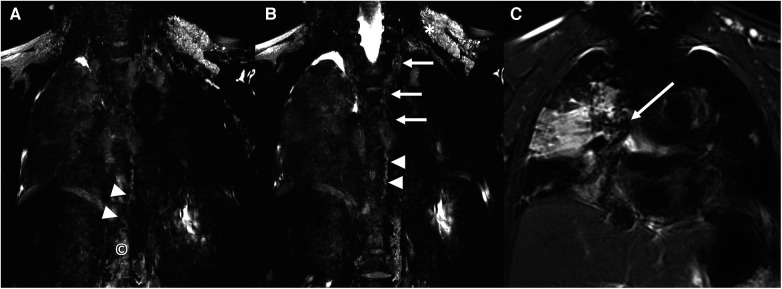
Coronal images from a three-dimensional, heavily T2-weighted MRI sequence (siemens magnetom vida 3 T). Cisterna chyli (**A**; ©) and the lower third of the thoracic duct (**A**,**B**; arrowheads) are demonstrated. The tubular aspect of the thoracic duct is lost in its middle and upper portions with an increased abnormal signal intensity at these levels (**B**; arrows). Increased abnormal signal intensity is also seen in the bilateral supraclavicular regions, in particular on the left side (**B**; *) extending into the mediastinum and with an interstitial pattern into the right lung (**A**–**C**), surrounding the right bronchus (**C**; arrow).

Primarily, a magnetic resonance lymphangiography (Siemens Magnetom Vida 3 T, Erlangen, Germany) showed a re-permeabilization of the TD just above the subdiaphragmatic clip and a pulmonary lymphatic perfusion syndrome type 4 mainly from the right upper lobe (Figure 1) (6, 7). A dynamic lymphography with possible embolization had to be interrupted due to the patient's ventilatory instability during the procedure.

A transthoracic echocardiogram (TTE) showed a preserved systolic function and ruled out a new-onset valvular regurgitation. The patient had persistent sinus rhythm without any arrhythmia.

To decompress the TD, we decided to adapt the original innominate vein turn-down procedure (3) by stenting the modified fenestration and plugging the distal innominate vein to relieve the pressure of the TD.

## 3 Transcatheter interventional procedure

Under sedation with spontaneous ventilation, we obtained access to the right and left internal jugular veins under ultrasound guidance. We administrated unfractionated heparin 100 UI/kg at the beginning of the procedure and controlled after 1 h the activated clotting time (ACT). To keep an ACT over 200 s during the procedure, we then checked the ACT every 30 min and gave a new bolus of unfractionated heparin as needed.

Hemodynamic measurements showed elevated cavopulmonary pressure (mean 22 mmHg) and a mean RA pressure of 8 mmHg. Angiography demonstrated no vena cava or pulmonary artery obstruction but a proximal stenosis at the level of the fenestration.

**Figure 2:**
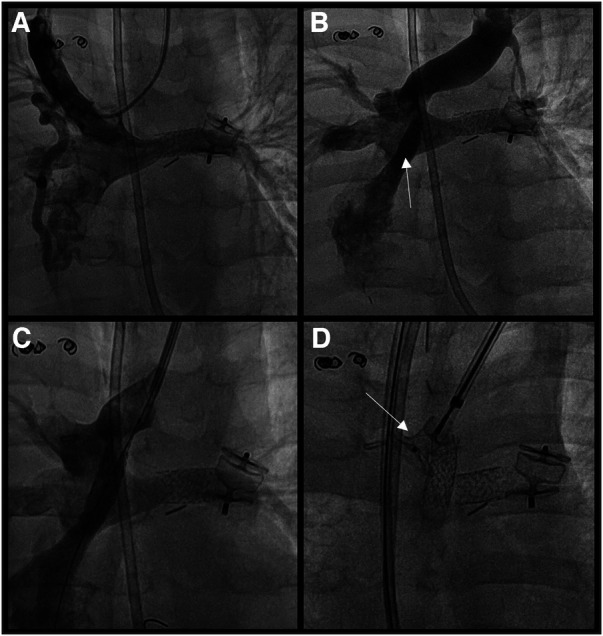
(**A**) native fontan circulation without any stenosis (**B**) permeable fenestration connecting the innominate vein to the single atrium (**C**) stenting of the fenestration (formula® 418 7/16 mm) (**D**) placement of ADO I® 10/8 mm to close the distal part of the innominate vein.

Through a 6 Fr sheath placed in the left internal jugular vein, a V-18 guidewire (Boston Scientific, Massachusetts, USA) was advanced from the innominate vein through the fenestration to the atrium. Under fluoroscopic control, a Formula® 418 7/16 mm balloon-expandable stent (Cook Medical, Indiana, USA) was placed in the proximal part of the fenestration and dilated at 8 bars for a diameter of 7 mm. Afterward, an Amplatzer Duct Occluder I (ADO I) 10/8 mm (Abbott, Illinois, USA) was placed at the distal part of the innominate vein (Figure 2). Before the device's release, a sudden and profound desaturation with severe bradycardia occurred. The patient stopped breathing and immediate cardiopulmonary resuscitation was initiated. As the ventilation was inadequate with a laryngeal mask, a tracheal intubation was placed. An emergency bronchoscopy was performed with the removal of several bronchial casts allowing adequate oxygenation and ventilation. This was followed by a prompt clinical improvement with restoration of the patient's initial cardiopulmonary parameters.

**Figure 3:**
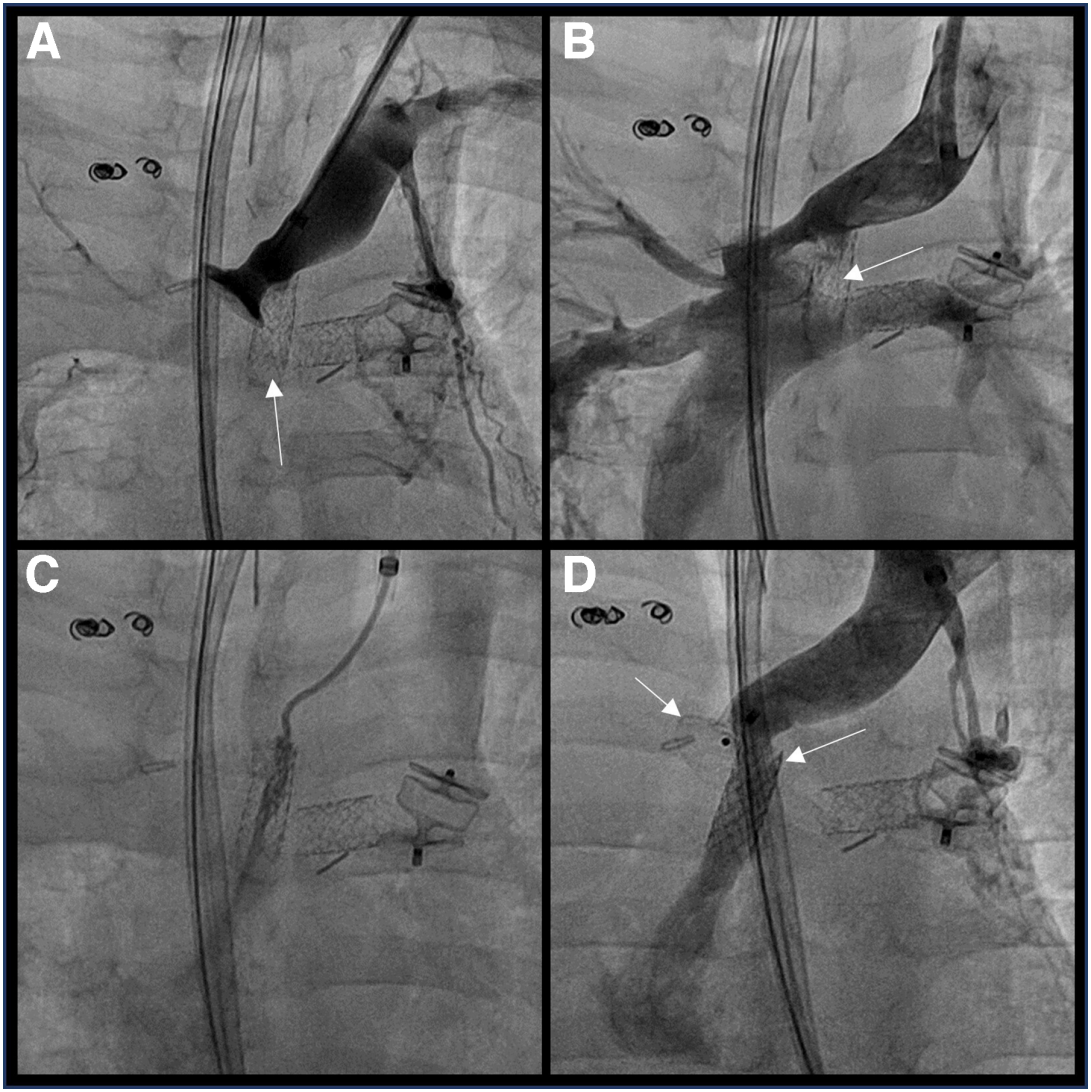
(**A**) objectivation of the fenestration thrombosis (**B**) removal of the ADO and reconfirmation of the fenestration thrombosis (**C**) local thrombolysis with alteplase (**D**) covered stent (gore viabahn® 7/19 mm) in the fenestration and ADO I® 10/8 mm closing the distal innominate vein creating a transcatheter innominate vein turn-down procedure.

Because of increasing cavopulmonary pressure over 25 mmHg, angiography in the innominate vein was repeated and demonstrated complete occlusion of the newly stented fenestration. After removing the ADO I to restore the permeability of the innominate vein to the superior vena cava and despite the risk of systemic embolism, we decided to perform local thrombolysis through an end-hole catheter at the entry of the fenestration with alteplase (1 mg/kg), and the fenestration could be permeabilized. The V-18 guidewire was placed in the RA again, the access sheath was changed to a Super Arrow-Flex 7 Fr 65 cm (Teleflex, Pennsylvania, USA), and a Viabahn 7/19 mm covered stent (Gore Medical, Delaware, USA) was deployed at the proximal end of the fenestration. The stented fenestration was free of obstruction with a mean gradient between the innominate vein and the RA of 3 mmHg. The ADO I was repositioned at the connection between the innominate vein and the superior vena cava. The device was released after a fluoroscopic check of the position and absence of occlusion of the stented fenestration (Figure 3). The final measurement demonstrates a mean pressure of 7 mmHg in the RA, 10 mmHg in the innominate vein, and an aortic SaO2 of 85%. At the superior vena cava and pulmonary artery levels, the mean pressure was 22 mmHg without stenosis.

Three hours after the transcatheter procedure and despite removal of bronchial casts by rigid fibroscopy, the patient was still in respiratory failure, reason why he was put on peripheral venoarterial extracorporeal membrane oxygenation (VA ECMO). Following a positive respiratory evolution, the patient was weaned from VA ECMO on day 6, extubated on day 8, and discharged on day 24 on oral anticoagulation (acenocoumarol) with no further cast expectoration.

**Figure 4:**
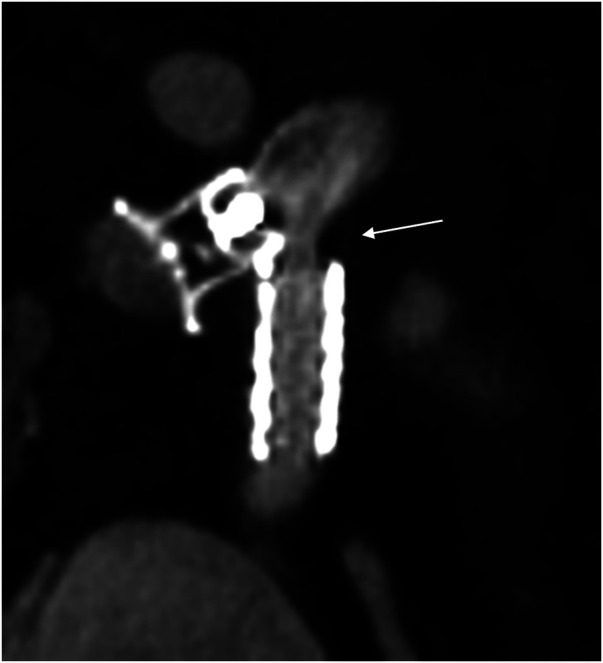
Unenhanced CT image of the ADO I® 10/8 mm closing the distal innominate vein and the covered stent (gore viabahn® 7/19 mm) in the fenestration with proximal stenosis of the fenestration (arrow).

At 6 months follow-up, the patient had two days of hospitalization due to new spontaneous bronchial cast expectoration. An unenhanced chest CT showed significant proximal stenosis of the fenestration (Figure 4). We proceeded with a new interventional procedure and got venous access in the left internal jugular and femoral veins under ultrasound guidance with the placement of 4F sheaths using the Seldinger technique. The initial hemodynamic assessment demonstrated an elevated pressure in the Fontan circulation of 19 mmHg. The innominate vein-RA fenestration was stenotic (Figure 4) with an invasive mean gradient of 9 mmHg (15 mmHg in the innominate vein vs. 6 mmHg in the RA). Through the 4 Fr sheath placed in the left internal jugular vein, a 0.035 exchange guidewire (Terumo, Tokyo, Japan) was advanced from the innominate vein through the fenestration into the atrium. With fluoroscopic control, a Formula® 535 7/20 mm balloon-expandable stent (Cook Medical, Indiana, USA) was implanted at the proximal anastomosis of the fenestration. The angiographic control showed a release of the stenosis, and the final hemodynamic assessment showed a reduction of the mean gradient between the innominate vein and RA to 3 mmHg. The patient did not experience any bronchial cast anymore, and transcutaneous saturation stayed stable at around 85%.

Triggered by an upper respiratory tract infection, the patient developed plastic bronchitis again 18 months after the initial transcatheter intervention. Chest CT and TTE ruled out restenosis of the fenestration. Despite desensitization strategies, 26 months later, the patient is still waiting for a heart transplant.

## 4 Discussion

The optimal fenestration of the Fontan circulation is a long-lasting debate in the literature (8, 9). Even if it has a similar hemodynamic effect, then venovenous collateral from the innominate vein to the coronary sinus, modifying the fenestration by connecting the innominate vein to the RA appendage, has multiple advantages. First, after weaning the cardiopulmonary bypass, it offers the possibility of assessing the hemodynamic situation and adapting the polytetrafluoroethylene graft for hemodynamic optimization. Second, it is easily viewable on TTE in a suprasternal view with an estimation of the mean gradient between systemic venous pressure and RA pressure. Third, it is easy to access from the left internal jugular vein for angioplasty, stenting, or closure. Fourth, we claim it reduces the risk of paradoxical embolic events because these emboli are more prone to arise from the lower body. Only small case series using this modified fenestration have been published (5, 10) but have suggested similar hemodynamic benefits as the fenestration between the extracardiac conduit and the atria.

As Smith et al. reported, other complex transcatheter strategies exist to decompress the TD into a lower-pressure chamber. These are of higher risk due to the creation of an extravascular connection and necessitate a complex 3D evaluation of the cardiac and mediastinal anatomy (11). Although the modified fenestration from the innominate vein to the RA was not intended for this purpose, it offers this new possibility of TD decompression through neither surgical procedure nor technically engaging extravascular connection. This procedure is technically more accessible and probably safer than the ones already described in the literature and can be done in all types of native anatomy. We strongly believe that the oxygenation with secondary hemodynamics difficulties encountered during and after the procedure are linked to the patient’s precarious respiratory status and not to the technique of the procedure. One reason for this is the significant improvement in respiratory status in the days following the procedure with no short-term relapse of the plastic bronchitis.

The median follow-up described by Smith et al. is 6 months with a range of 1–20 months, with improvement or resolution of the lymphatic problem in about two-thirds of the patients (11). Unfortunately, despite an initial complete relapse of the symptoms, the follow-up, in our case, showed a reappearance of the bronchial casts at 6 months secondary to fenestration's restenosis and a second relapse at 18 months, triggered by an airway tract infection. The long-term relapse of the symptoms by surgical or interventional TD decompression is still uncertain. More efforts are needed to better diagnose and stratify the patients and identify those likely to achieve good long-term outcomes without symptom relapse.

## 5 Conclusion

Although the modified fenestration from the innominate vein to the RA was not intended for this purpose, it offers this new possibility of TD decompression without surgical procedures. This rescue intervention isolates the innominate vein from the Fontan circulation by decreasing the pressure of the TD. This procedure seems technically more accessible and probably safer than the ones already described in the literature and could be done in all types of native anatomy. For this type of intervention, further evaluations and extended follow-up are required to identify the patients likely to achieve good long-term outcomes without symptom relapse.

## Data Availability

The original contributions presented in the study are included in the article/Supplementary Material, further inquiries can be directed to the corresponding author.

## Supplementary material

Supplementary Figure 1 Patient timeline.
