# Supplementary figures and images for: Case Report: Transcatheter interventional procedure to innominate vein turn-down procedure for failing fontan circulation
Source: Front Pediatr. 2024 Feb 6;12:1341443. doi: 10.3389/fped.2024.1341443 (PMC10876887; doi:10.3389/fped.2024.1341443)

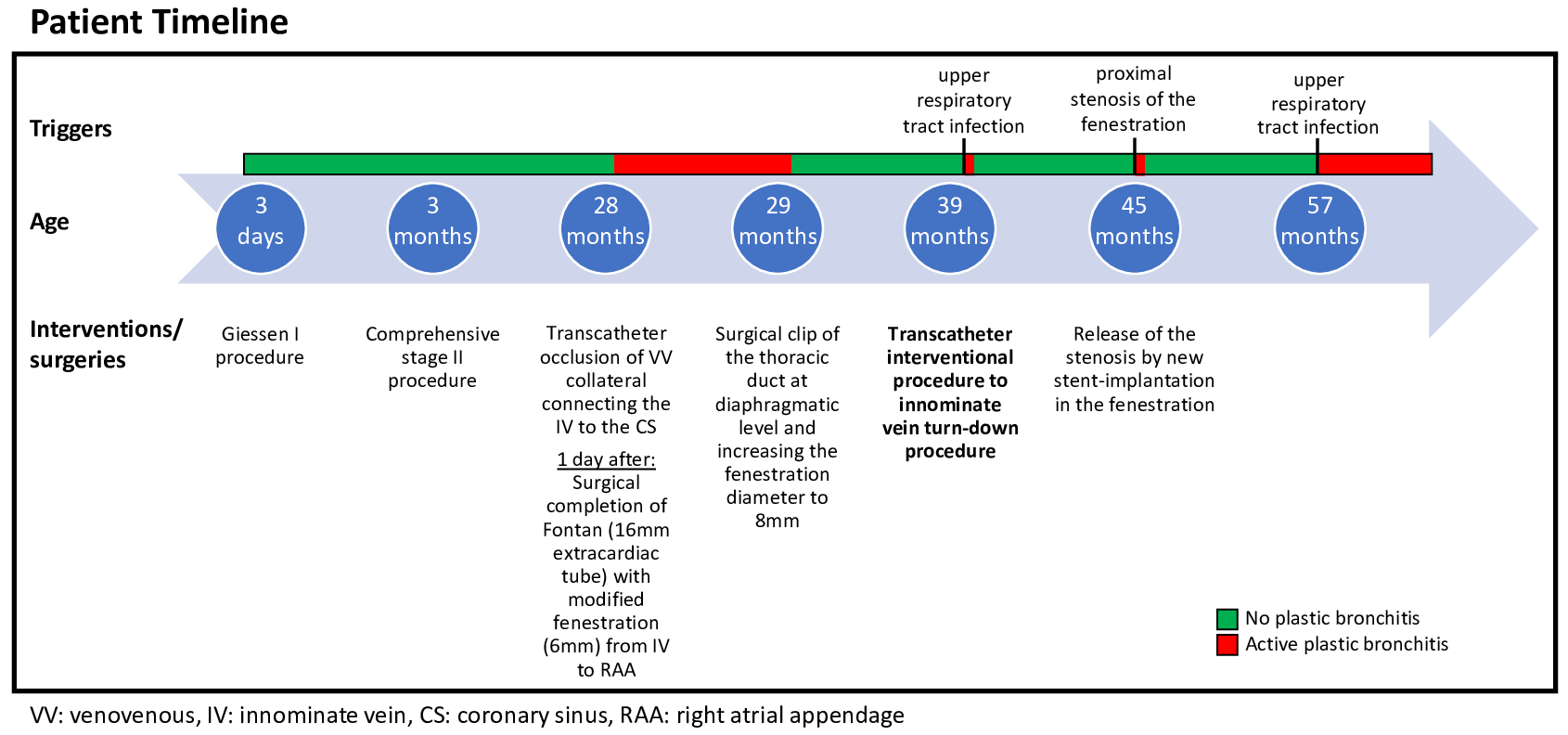

Supplement: Supplementary Figure 1 [file Image1.tif]
